# Urticarial vasculitis induced by OTC diet pills: a case report

**DOI:** 10.1186/s40413-015-0059-y

**Published:** 2015-04-16

**Authors:** Iván Chérrez Ojeda, Enrique Loayza, Leonardo Greiding, Juan Carlos Calderón, Annia Cherrez, Farid Adum

**Affiliations:** School of Medicine, Universidad Espíritu Santo, Guayaquil, Ecuador; RespiraLab Research Group, Hospital-Clínica Kennedy, Guayaquil, Ecuador; Centro Dermatológico Loayza, Guayaquil, Ecuador; Instituto Argentino de Alergia e Inmunología, Buenos Aires, Argentina; School of Medicine, University of Heidelberg, Heidelberg, Germany

**Keywords:** Urticarial Vasculitis, OTC diet pills, Weight loss

## Abstract

**Background:**

Urticarial Vasculitis (UV) is in most of the cases idiopathic; however it has been associated with several conditions and drugs. Over the counter (OTC) diet pills are widely available, even on-line, but they are rarely regulated by pharmaceutical control.

**Case presentation:**

We present the case of a 35-year-old female patient suffering of pruriginous and painful wheals more than 1 cm in diameter, with a burning sensation. The eruption lasted more than 24 hours and was accompanied by angioedema, headache and myalgia. No remarkable medical history was found, except for previous intake of OTC diet pills. UV diagnosis was confirmed by the skin biopsy of a lesion.

**Conclusion:**

OTC diet pills are widely available worldwide, and due to its widespread use, allergologists and dermatologist should be able to recognize symptoms and lesions of cutaneous vasculitis, which may be under reported.

## Background

The majority of cases of Urticarial Vasculitis (UV) are idiopathic; however it has been associated with many different disorders such as infections, connective tissue diseases, malignancies and also drugs [1]. UV lesions persist for 24 hours or more and can cause pruritus but are more commonly painful, with stinging or burning sensation, and leave a faint residual hyperpigmentation. Extra-cutaneous manifestations include arthralgia, abdominal pain, obstructive lung disease, nephritis, and uveitis [2]. Histological criteria for the diagnosis of UV include nuclear debris or fibrin deposits, with or without extravasation of red blood cells [3].

A large number of weight-loss pills are available (OTC). Even more options can be acquired on line. Some of them report dangerous side effects but, being marketed as a supplement rather than a drug, these pills are not subject to rigorous standards of quality control by an agency.

## Case presentation

**Figure 1:**
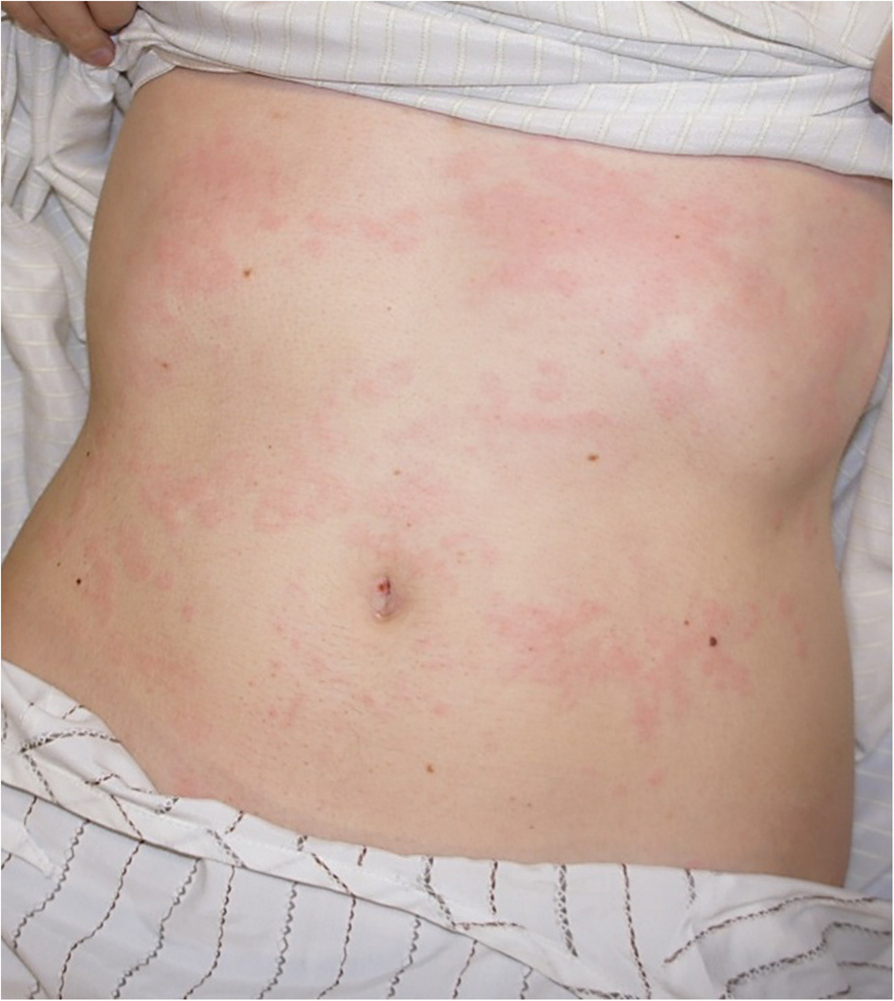
**Wheals and erythematous maculae in trunk, back and abdomen were found.**

**Figure 2:**
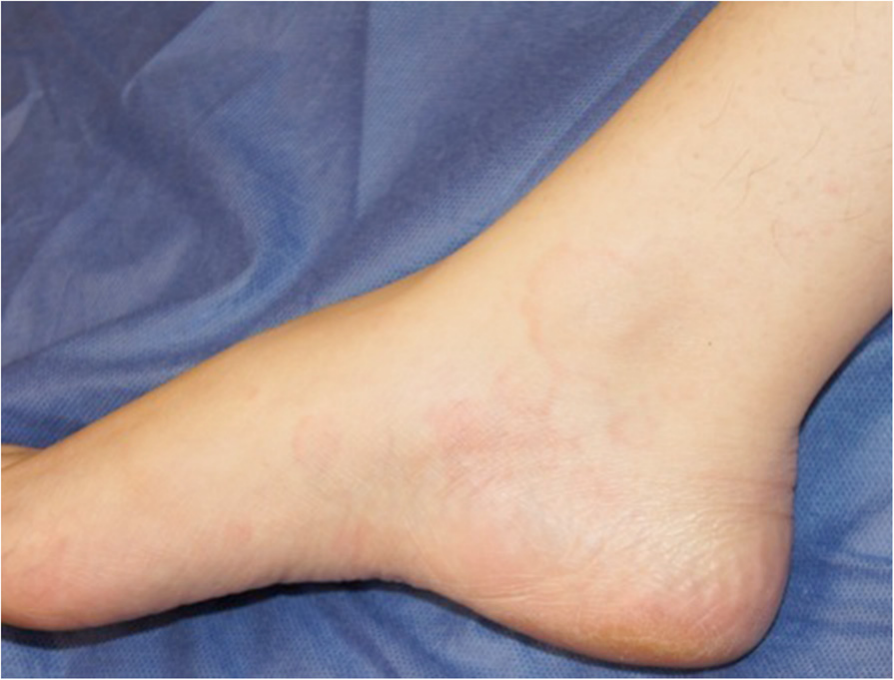
**Wheals and erythematous maculae also can be found in ankle.**

A 35 year old woman with a history of intermittent rhinitis presented with a 5 day history of urticaria, fever, headaches, myalgia and arthralgia. She developed hives and erythematous maculae in her trunk, back, abdomen and limbs (Figures 1, 2); they were pruriginous and painful, with a burning sensation, and had more than 1 cm in diameter. The lesions lasted more than 24 hours, altering the pigmentation of the area. Angioedema was present in hands, fingers, ankles and feet, and left no pitting.

Severe headaches and myalgia impaired the patient’s ability to conduct her daily routine and fever spiked as high a 39.5 C.

The patient had been taking OTC diet pills eighteen days before, and six days previously consulted for dehydration and violaceous hives in her palms; after that she suspended the pills.

Physical examination revealed large confluent areas of wheals and maculae in her trunk, limbs and abdomen, scored 6/10 in intensity of the pruritus by the patient, whom also complained of muscular pain. The rest of the examination was unremarkable. She had a normal hemoglobin concentration and lymphopenia. The C-reactive protein concentration was 7.5 mg/L (normally <5 mg/L); complement components, liver function test, urea and creatinine, plasma sodium, glucose and thyroid hormones and antibodies were within normal ranges. Antinuclear antibodies (ANA) were not detectable in blood.

The skin biopsy of one lesion was performed, in which a slight alteration of vacuolar interface was observed, the papillary dermis presented edema and there was a moderate superficial and perivascular infiltrate of lymphocytes and eosinophils with focal fibrin deposits in blood vessel walls and extravasation of erythrocytes. After confirmation of the diagnosis of UV, the patient was prescribed with oral corticosteroids and antihistamines, achieving rash remission. Due to persistence of headaches, fever and myalgia, hydroxychloroquine was added to the treatment, with remission of all the symptoms one month later.

### Discussion about UV

UV is a condition that usually presents itself with wheals in the proximal areas of the limbs and in the trunk, often more painful than pruriginous, with a duration of symptoms of more than 24 hours, a violaceous shade to the lesions, and residual pigmentation after resolution [4]. Two forms have been described: hypo-complementemic UV and normo-complementemic UV. The hypo-complementemic form may as well manifest with or without systemic symptoms [2].

UV is in most of the cases idiopathic, but there is a wide variety of conditions, such as infections, hematologic disorders, physical urticaria, exercise, drugs, etc., that have been associated with this disease. The most commonly linked disorders are connective tissue diseases, particularly SLE and Sjögren’s syndrome. UV is thought to represent a type III hypersensitivity reaction due to the circulation immune complexes that may be found in up to 75% of patients [5].

UV lesions damage the capillary and post-capillary venules. Histopathological findings include perivascular neutrophilic or lymphocytic infiltrates with fibrinoid necrosis, evidence of fully developed leukocytoclastic vasculitis with injury and swelling of the nuclei of endothelial cells of postcalpillary venules and extravasation of erythrocytes [6].

Lymphocytes can predominate or be the only cell observed in older vasculitic lesions. Fibrinoid changes or necrosis and particularly thrombosis are less common in UV than in the fully developed lesions of palpable purpura. There is usually little vessel wall disruption and only a mild degree of perivascular infiltrate, without red blood cell extravasation, in young UV lesions. Such lesions can be difficult to distinguish from true urticarial [2].

In our case report, the ingredients in the OTC diet pills were: synephrine HCL, synthetic 99% guggulsterones, thyroid stimulating matrix, Yohimbine HCL and phenylamine.

The unregulated use of synephrine in dietary supplements is arising controversy in the scientific community, namely regarding to its safety and efficacy when it is used in weight-loss products [7]. It is well known that the occurrence of severe cardiovascular toxicity related to the consumption of synephrine-containing products determined the temporary prohibition of synephrine in dietary products in Canada [8].

Yohimbine substantially increases the incidence of adverse drug side effects such as: gastrointestinal discomfort (46%), tachycardia (43%), anxiety/agitation (33%), and hypertension (25%). Yohimbine exposures were associated with a significantly higher number of severe adverse reactions to drugs, and were more likely to require management at a health-care facility (odds ratios [95% CIs] were 5.81 [4.43 to 7.64] and 2.35 [1.82 to 3.04], respectively [9]. Guggulsterones have been associated with fulminant hepatic failure, requiring emergency liver transplants [10].

Although all of these ingredients have reported adverse side effect, this may be the first report of them inducing vasculitis. We believe that the development of UV might have been induced by the use of diet OTC pills, based on the temporal relationship between the intake of the pills and the appearance of the dermatological lesions and systemic manifestations in the lack of another trigger. The histologically proven vasculitis and finally the reversal of the clinical signs after discontinuation of the OTC diet pills also suggest an association.

UV is a manifestation of inflammatory injury of capillaries and post-capillary venules in the skin. The ingredients found in these OTC diet pills have the capacity of inducing IgG antibodies against the collagen-similar regions of C1q to form immune complexes, initiating an inflammatory cascade that leads to mast cell degranulation. The substances released thereby result in increased vascular permeability causing urticaria and/or angioedema [2].

In our center, we have seen several patients who were using the same diet pills and developed urticaria, but ethical considerations prevent the conduction of a challenge test to confirm the association, due to the unpredictability of side effects that may occur on patients.

## Conclusions

Several weight loss pills are freely available both OTC and online. Most have not been proved effective and some may contain dangerous substances that can cause life-threatening side effects. UV has been reported in association with a large amount of drugs, but this might be the first report to link UV with use of OTC diet pills.

Given the widespread use of OTC diet pills, allergologists and dermatologists should be able to recognize cutaneous vasculitis, which may be under reported.

## Consent

Written informed consent was obtained from the patient for publication of this case report and accompanying images. A copy of the written consent is available for review by the Editor-in-Chief of this journal.

## Abbreviations

OTC: Over the counter
UV: Urticaria vasculitis
